# Mathematical correction of the effects of storage time and gas contamination on blood sample measurements

**DOI:** 10.3389/fmed.2025.1630871

**Published:** 2025-09-25

**Authors:** Bahareh Nevirian, Lars Pilegaard Thomsen, Steen Kåre Fagerberg, Jette Nybo, Mette Krogh Pedersen, Kjeld Asbjørn Jensen Damgaard, Lisha Shastri, Søren Risom Kristensen, Stephen Edward Rees

**Affiliations:** ^1^Respiratory and Critical Care (Rcare) Group, Aalborg University, Aalborg, Denmark; ^2^Department of Clinical Medicine, Aalborg University, Aalborg, Denmark; ^3^Anesthesia and Intensive Care Unit, Aalborg University Hospital, Aalborg, Denmark; ^4^Department of Clinical Biochemistry, Aalborg University Hospital, Aalborg, Denmark

**Keywords:** acid-base, mathematical modeling, analysis delay, gas contamination, venous blood sampling

## Abstract

**Objective:**

Analysis delay and gas contamination can affect the accuracy of blood measurements. This study uses a mathematical model of blood acid-base chemistry and gas in the sample tubes to calculate values of pH, partial pressures of carbon dioxide (pCO_2_), partial pressure of oxygen (pO_2_), oxygen saturation in whole blood (SO_2_), glucose, and lactate at sample time from measurements with delayed analysis and gas contamination.

**Methods:**

Data were analyzed from two published studies. Study 1: Samples were obtained from 30 critically ill patients in standard blood gas syringes and analyzed after 0, 36, 54, 72, 90, 108, 126, 144, 162, and 180 min. Study 2: Samples were taken from 20 healthy participants in standard blood gas syringes and vacuum tubes (2 mL and 4 mL) and analyzed after 0, 20, and 90 min. Calculated values from the mathematical model were compared to measured values at sample time.

**Results:**

For delays of up to 90 min, the accuracy (mean) and precision (standard deviation (SD)) values calculated at the sample time using syringes and 4-mL vacuum tubes remained within clinically acceptable limits when compared to measured values, with the exception of SO₂ in vacuum tubes. Values represent the mean difference ± standard deviation between calculated and measured values. For syringes, the results were as follows: pH = −0.004 ± 0.011, pCO₂ = 0.08 ± 0.18 kPa, pO₂ = 0.05 ± 0.34 kPa, SO₂ = 0.39 ± 2.21%, glucose = 0.07 ± 0.35 mmol/L, and lactate = 0.13 ± 0.22 mmol/L. For 4-mL vacuum tubes, the results were as follows: pH = 0.006 ± 0.007, pCO₂ = −0.07 ± 0.11 kPa, pO₂ = −0.37 ± 0.34 kPa, SO₂ = −7.79 ± 4.95%, glucose = 0.01 ± 0.11 mmol/L, and lactate = −0.00 ± 0.20 mmol/L. In addition, 2-mL vacuum tubes had poorer accuracy and precision values than syringes and 4-mL vacuum tubes in a subset of cases.

**Conclusion:**

This study has shown that a mathematical model can accurately and precisely calculate blood values at sample time, even following delayed analysis, using both standard blood gas syringes and selected vacuum tubes. This method may have clinical applications in improving the logistics of blood sampling and analysis.

## Introduction

Anaerobic metabolism in erythrocytes causes biochemical changes that, over time, affect measured values of acid-base, oxygenation, and metabolism in blood samples. Consequently, the time passed between blood collection and analysis is an important factor influencing the validity of these measurements (1–9). In addition to the timing of analysis, the type of tube or syringe used for blood collection can influence the validity of measurements. Sampling with vacuum tubes that contain residual air after blood collection results in altered partial pressures of carbon dioxide (pCO₂) and oxygen (pO₂) in the blood due to the diffusion of CO₂ and O₂ between the blood and the residual air in the tube (10).

In previous studies (11, 12), we used a physicochemical mathematical model to simulate changes in acid-base status, oxygenation, electrolytes, and metabolic status under conditions of delayed analysis and gas contamination. In those studies, the model was used in the forward direction—from the sample time to the analysis time—to describe how blood values change with delay and gas exposure. However, the main challenge in clinical practice is not predicting forward, but rather being able to back-calculate the values at the sample time from blood sample measurements affected by delayed analysis and/or gas contamination. In the present study, we therefore applied an inverse use of the model, calculating values at the sample time from the values obtained at the analysis time. This mathematical inversion has not been implemented in the previous study (11, 12) and represents the essential novelty of our approach. Moreover, by calculating the accuracy and precision of these back-calculated values for different delay durations, we provide a practical framework that allows clinicians and researchers to determine the maximum acceptable delay for reliable blood gas analysis. This practical application extends the utility of the physicochemical mathematical model beyond the description of changes toward the correction of delayed and/or gas-contaminated blood samples stored in either standard blood gas syringes or vacuum tubes.

## Method

**Figure 1:**
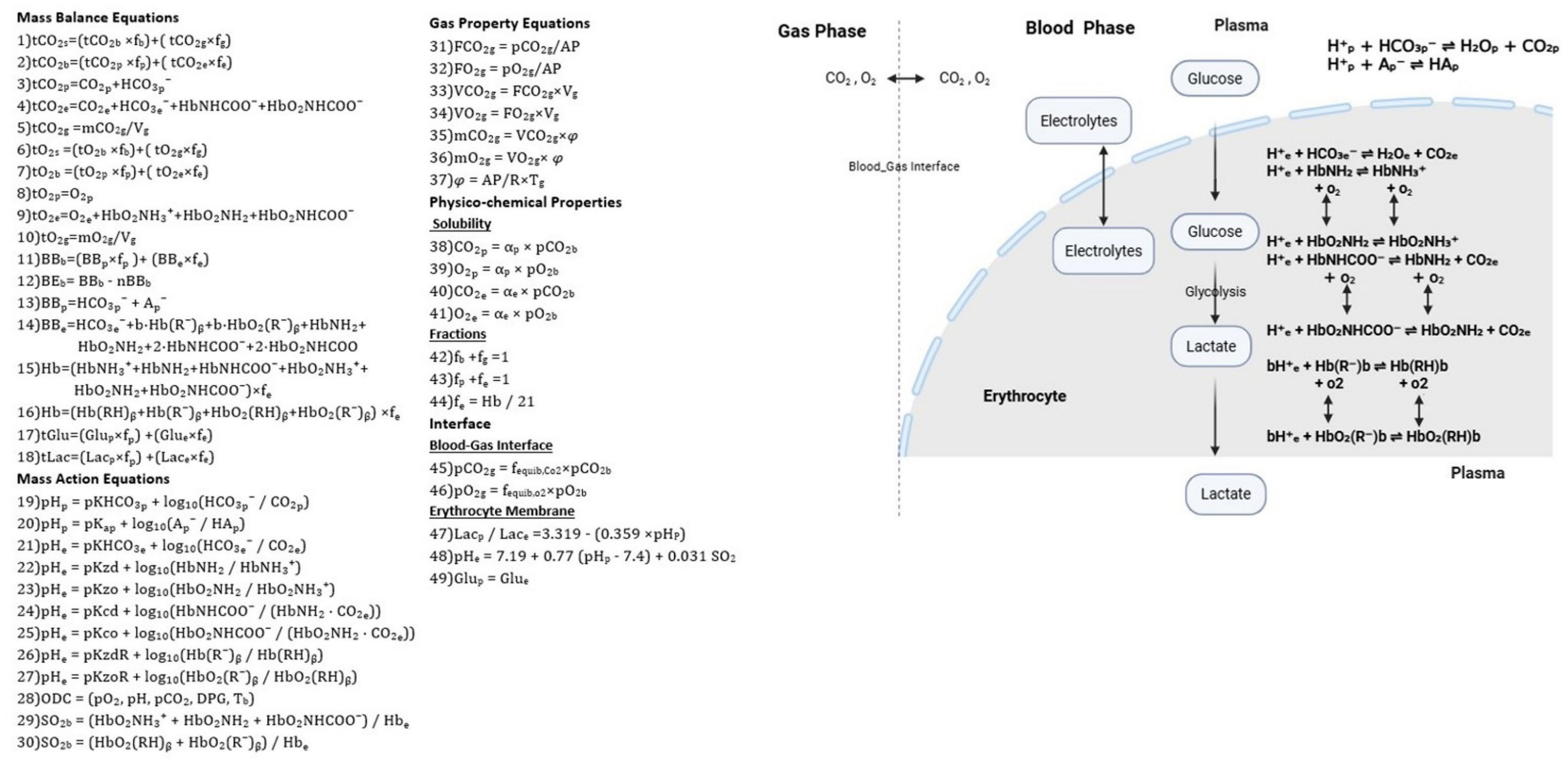
Equations and physicochemical processes describing the blood and gas phases (see equation numbers in the figure). Mass balance equations (1–18), mass action equations (19–30), gas properties (31–37), physicochemical properties (38–44), and the interface between gas and blood and between the plasma and erythrocyte (45–49).

The applied mathematical model describes three compartments that may exist in a blood sample: (1) the gas phase, i.e., a significant gas or air bubble occupying a fraction of the sample tube, as occurs in vacuum tubes; (2) the plasma fraction of blood; and (3) the erythrocyte fraction of blood. Equations were formulated to describe mass balance, mass action, and the physicochemical properties of these compartments, as well as the interfaces between gas and blood and between plasma and erythrocytes, as illustrated in Figure 1. Figure 1 provides a schematic overview of the mathematical model, including key equations and compartmental interactions.

Detailed explanations of the equations and the corresponding table of abbreviations have been moved to the Supplementary material in accordance with the journal’s guidelines. Readers are encouraged to consult this material to fully understand the methodology before proceeding.

In essence, the model represents the dynamic exchange and equilibration of gases (O₂ and CO₂), ions, and metabolites between the gas, plasma, and erythrocyte compartments of a blood sample. By incorporating diffusion processes, buffering of hydrogen ions, and hemoglobin (Hb) binding reactions, it simulates the alterations in measured values that occur during storage or in the presence of residual gas in the blood tube. In this study, the model is applied in an inverted manner to back-calculate the values at the sample time from those obtained at the analysis time.

### Model simulation

The mathematical model in Figure 1 was applied to simulate the plasma values of pH (pH_p_), partial pressures of carbon dioxide (pCO_2p_) and oxygen (pO_2p_), glucose (Glu), lactate (Lac), and oxygen saturation in whole blood (SO_2b_) at the sample time from measured values at the analysis time. This was performed for the data of two previously conducted studies.

Study 1: Venous blood samples were obtained from 30 critically ill patients admitted to an intensive care unit (ICU), collected in standard blood gas syringes, and analyzed after 0, 36, 54, 72, 90, 108, 126, 144, 162, and 180 min.

Study 2: Peripheral venous blood samples were taken from 20 healthy participants, collected into both standard blood gas syringes and vacuum tubes (2 mL and 4 mL), and analyzed after 0, 20, and 90 min. However, for the simulations presented here and the analysis results shown in the “Results” section, only the vacuum tube data at 20 and 90 min were included. Data from standard blood gas syringes at 90 min in Study 2 were not included in this analysis, as our focus in this study was specifically on data from vacuum tubes. The vacuum tubes used in this study were identical in total volume but designed to draw either 2 mL or 4 mL of blood, leaving a defined volume of the remaining air above the fill line. This remaining air, determined by the manufacturer’s design, was included in the model to reflect typical clinical conditions and potential gas exchange during storage.

In both studies, blood samples were stored at room temperature prior to analysis, and the applied model accounted for the metabolic effects of erythrocyte changes occurring at room temperature.

**Figure 2:**
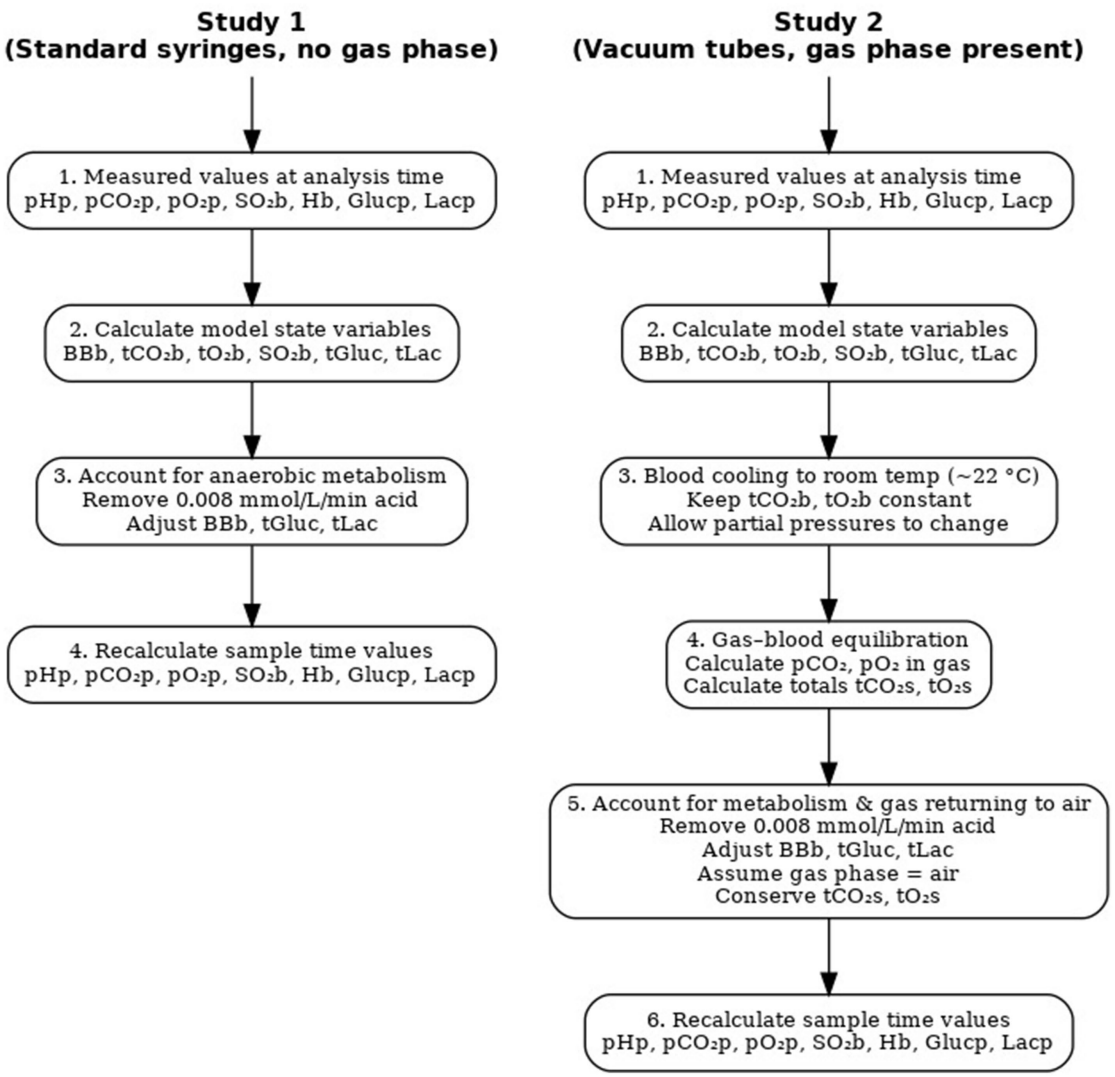
Model simulation strategy.

Study 1 and Study 2 differed. In Study 1, blood samples were taken in standard blood gas syringes (PICO safe heparin syringes, Radiometer, Denmark) with no gas phase, and observable changes in samples were attributed primarily to the metabolic effect of erythrocytes. In Study 2, blood samples were collected in vacuum tubes (VACUETTE^®^ 454001, 454088, Greiner Bio-One, Kremsmünster, Austria), allowing observable changes in the samples to reflect a mix of gas diffusion between the gas phase and blood, as well as erythrocyte metabolism. Accordingly, the simulations performed for each of these two studies differ but use the same computational model illustrated in Figure 1. These simulations are described here and illustrated by the flowchart in Figure 2.

In Study 1, the values of pH_p_, pCO_2p_, pO_2p_, SO_2b_, Hb, Glu, and Lac measured using a blood gas analyzer (ABL 835 FLEX, Radiometer) at analysis time were used to calculate the state variables from the model: blood buffer base (BB_b_), total concentration of CO₂ (tCO_2b_) and O₂ (tO_2b_) in blood, SO_2b_, and total concentration of Glu and Lac (tGluc and tLac). For Study 1, values of concentrations of O_2_ and CO_2_ in the gas compartment were set to zero, reflecting blood sampling in standard blood gas syringes without air contamination. This modeling assumption implies that no significant gas phase was present, and thus, gas exchange between blood and a gas compartment was considered negligible. Consequently, all equations numbered 1, 6, 31–37, 42, and 45–46 related to the gas phase were not applied.

Simulating the conditions at the sample time based on values measured at the analysis time requires adjusting for the acid produced by anaerobic erythrocyte metabolism. To do so, the acid added as part of erythrocyte metabolism due to sampling delay was removed. This removal was performed by reducing the base excess (BE) and buffer base (BB) of whole blood by an acid production rate of 0.008 mmol/L/min, taking into account the duration of the delay, removing an equivalent amount of lactate, and adding half the amount of glucose to account for usual anaerobic metabolism. The value of 0.008 mmol/L/min was the value obtained in Nevirian et al. (11). This modifies the values of the state variables for the mathematical model (Figure 1), which can then be re-solved to calculate all model variables, including pH_p_, pCO_2p_, pO_2p_, SO_2b_, Glu, and Lac_._

In Study 2, calculation from analysis time to sample time requires accounting for both anaerobic metabolism and CO₂ and O₂ distribution between blood and gas compartments. Values measured in blood were reported at 37 °C. However, during the delay before analysis, the blood in the vacuum tube cooled to approximately room temperature. Therefore, the first step was to mathematically convert the measured values to those corresponding to the lower temperature. This conversion was performed at 22 °C using equations from the mathematical model in Figure 1, mainly by adjusting the oxygen dissociation curve for temperature effects (13), while assuming a constant total O₂ concentration in the blood (Equation 28).

Following this cooling, the resulting simulated values of pCO_2_ and pO_2_ in blood, i.e., those calculated at 22 °C, were assumed to be in partial equilibrium with those in the gas phase according to equations 45 and 46 of the model. Calculating pCO_2_ and pO_2_ in the gas phase following this method allows for calculating the total concentration of CO_2_ and O_2_ in the whole system, which includes both the blood and gas phase (tCO₂_s_ and tO₂_s_), according to equations 1 and 6. Due to mass conservation in this closed tube, these totals remain the same at both sample time and analysis time and can therefore be used—along with two other pieces of information—to enable a complete solution of the mathematical model to calculate all values at sample time. The required information is the composition of the gas phase and the rate of anaerobic metabolism. We assumed that the gas in the vacuum tube at sample time was air, with pO₂ = 21 kPa and pCO₂ = 0.04 kPa, and that the effects of anaerobic metabolism could be calculated, as in the previous study, by modifying blood BE/BB for an acid production rate of 0.008 mmol/L/min.

Some data from Study 2 were excluded from the analysis. After correcting the measured values of pCO₂ and pO₂ at analysis time to 22 °C, occasionally the calculated partial pressure of oxygen in blood remained higher than in air, i.e., >21 kPa. Since all subjects breathed ambient air, pO₂ values above this threshold were not physiologically possible and indicate numerical error rather than true measurement. In these cases, the model attempted to reverse the gas exchange that occurred during storage; however, the result indicated that oxygen remained in the blood at a higher level than that attained under atmospheric breathing conditions. As this issue can be identified within the method, it reflects a limitation in applicability rather than introducing errors. Three data points were excluded due to this, representing 4% of the total calculations performed.

### Presentation of results

Results from both Study 1 and Study 2 are presented in two complementary formats: tables and plots.

Tables 1, 2 summarize the mean differences and standard deviations (SD) of the differences for six variables—pH, pCO₂, pO₂, SO₂, Glu, and Lac—for both pre-correction (before applying the model) and post-correction (after applying the model) analyses. For pre-correction, the differences were calculated as the measured baseline values (time 0) minus the measured values from samples analyzed at specific time points: 36, 54, 72, 90, 108, 126, 144, 162, and 180 min for Study 1 and 20 and 90 min for 4-mL and 2-mL vacuum tubes in Study 2. For post-correction, the differences were calculated as the baseline values minus the values back-calculated by the mathematical model at the sample time. The model used measurements from the later analysis times to correct for delayed analysis in Study 1 and both delayed analysis and gas contamination in Study 2, thereby calculating the original blood values at the time the sample was taken.

**Table 1:** Mean (±SD) of the differences for both pre-correction and post-correction analyses in standard blood gas syringes from Study 1.

Time min	pH pre	pH post	pCO_2_ kPa pre	pCO_2_ kPa post	pO_2_ kPa pre	pO_2_ kPa post	SO_2_% pre	SO_2_% post	Glu mmol/L pre	Glu mmol/L post	Lac mmol/L pre	Lac mmol/L post
36	0.009 (0.006)	−0.004 (0.007)	−0.05 (0.25)	0.12 (0.23)	−0.17 (0.33)	−0.10 (0.34)	−1.04 (2.52)	−1.06 (2.53)	0.09 (0.21)	−0.05 (0.22)	−0.25 (0.11)	0.06 (0.12)
54	0.013 (0.007)	−0.006 (0.009)	−0.13 (0.17)	0.12 (0.19)	−0.11 (0.24)	0.00 (0.25)	−0.52 (1.70)	−0.54 (1.80)	0.24 (0.27)	0.03 (0.27)	−0.39 (0.16)	0.09 (0.16)
72	0.019 (0.009)	−0.007 (0.011)	−0.22 (0.19)	0.13 (0.21)	−0.17 (0.32)	−0.02 (0.32)	−0.12 (1.95)	−0.15 (1.95)	0.32 (0.26)	0.04 (0.27)	−0.51 (0.20)	0.12 (0.20)
90	0.027 (0.008)	−0.004 (0.011)	−0.34 (0.21)	0.08 (0.18)	−0.14 (0.33)	0.05 (0.34)	0.42 (2.21)	0.39 (2.21)	0.43 (0.34)	0.07 (0.35)	−0.67 (0.22)	0.13 (0.22)
108	0.032 (0.011)	−0.006 (0.013)	−0.39 (0.22)	0.13 (0.19)	−0.15 (0.45)	0.07 (0.46)	0.82 (2.59)	0.78 (2.59)	0.45 (0.34)	0.03 (0.35)	−0.80 (0.30)	0.15 (0.29)
126	0.040 (0.012)	−0.004 (0.017)	−0.48 (0.19)	0.13 (0.21)	−0.17 (0.41)	0.10 (0.45)	1.27 (2.74)	1.23 (2.73)	0.55 (0.30)	0.05 (0.31)	−0.95 (0.27)	0.17 (0.26)
144	0.049 (0.016)	−0.001 (0.019)	−0.57 (0.33)	0.11 (0.31)	−0.21 (0.51)	0.09 (0.53)	1.26 (2.87)	1.21 (2.87)	0.66 (0.34)	0.09 (0.34)	−1.18 (0.35)	0.09 (0.34)
162	0.053 (0.016)	−0.002 (0.018)	−0.64 (0.30)	0.13 (0.25)	−0.25 (0.54)	0.08 (0.55)	1.71 (3.43)	1.66 (3.43)	0.73 (0.36)	0.08 (0.36)	−1.31 (0.33)	0.12 (0.33)
180	0.060 (0.019)	−0.001 (0.023)	−0.67 (0.32)	0.17 (0.38)	−0.24 (0.60)	0.13 (0.61)	1.86 (3.25)	1.80 (3.25)	0.84 (0.35)	0.12 (0.36)	−1.51 (0.41)	0.08 (0.40)

min = minutes; pre = pre-correction analyses; post = post-correction analyses.

**Table 2:** Mean (±SD) of the differences for both pre-correction and post-correction analyses in 4-mL and 2-mL vacuum tubes from Study 2.

Time min	pH pre	pH post	pCO_2_ kPa pre	pCO_2_ kPa post	pO_2_ kPa pre	pO_2_ kPa post	SO_2_% pre	SO_2_% post	Glu mmol/L pre	Glu mmol/L post	Lac mmol/L pre	Lac mmol/L post
20 (V4mL)	0.030 (0.014)	0.007 (0.005)	−0.38 (0.23)	−0.05 (0.11)	−4.94 (4.50)	−0.25 (0.23)	−28.73 (12.25)	−7.20 (3.68)	0.11 (0.08)	0.03 (0.08)	−0.25 (0.13)	−0.06 (0.13)
90 (V4mL)	0.048 (0.011)	0.006 (0.007)	−0.67 (0.21)	−0.07 (0.11)	−4.54 (3.69)	−0.37 (0.34)	−29.43 (12.11)	−7.79 (4.95)	0.35 (0.11)	−0.01 (0.11)	−0.84 (0.20)	0.00 (0.20)
20 (V2mL)	−0.014 (0.023)	0.010 (0.034)	0.85 (0.30)	−0.04 (0.55)	−16.89 (2.44)	−0.32 (1.83)	−50.01 (24.39)	−3.87 (17.42)	0.10 (0.11)	0.02 (0.11)	−0.30 (0.14)	−0.09 (0.14)
90 (V2mL)	0.000 (0.027)	0.021 (0.030)	0.64 (0.43)	−0.28 (0.52)	−17.26 (2.36)	−1.33 (3.96)	−49.99 (24.48)	−12.46 (18.64)	0.36 (0.14)	0.00 (0.14)	−0.94 (0.25)	−0.07 (0.24)

V4mL and V2mL indicate 4-mL and 2-mL vacuum tubes, respectively. Min = minutes; pre = pre-correction analyses; post = post-correction analyses.

Corresponding plots visualize the post-correction results at these same time points. Additionally, Bland–Altman plots were constructed for each variable across all patients/subjects and are provided in the Supplementary material to evaluate agreement between measured and model-calculated values at sample time (14). The data summarized in Tables 1, 2 are also visualized as error bars showing mean differences and SDs for both pre- and post-correction in the Supplementary material.

## Results

As shown in Tables 1, 2, applying the model resulted in a reduction of mean differences (bias) across all time points, except for SO₂ in Study 1, for which the reason will be discussed in the “Discussion” section. The bias between measured values at sample time and measured values at different analysis time points decreased after correction (post-correction), resulting in values closer to the actual measured values at sample time compared to pre-correction. This indicates that the model effectively reduces systematic errors caused by delayed analysis and/or gas contamination in blood samples in both Study 1 and Study 2.

**Figure 3:**
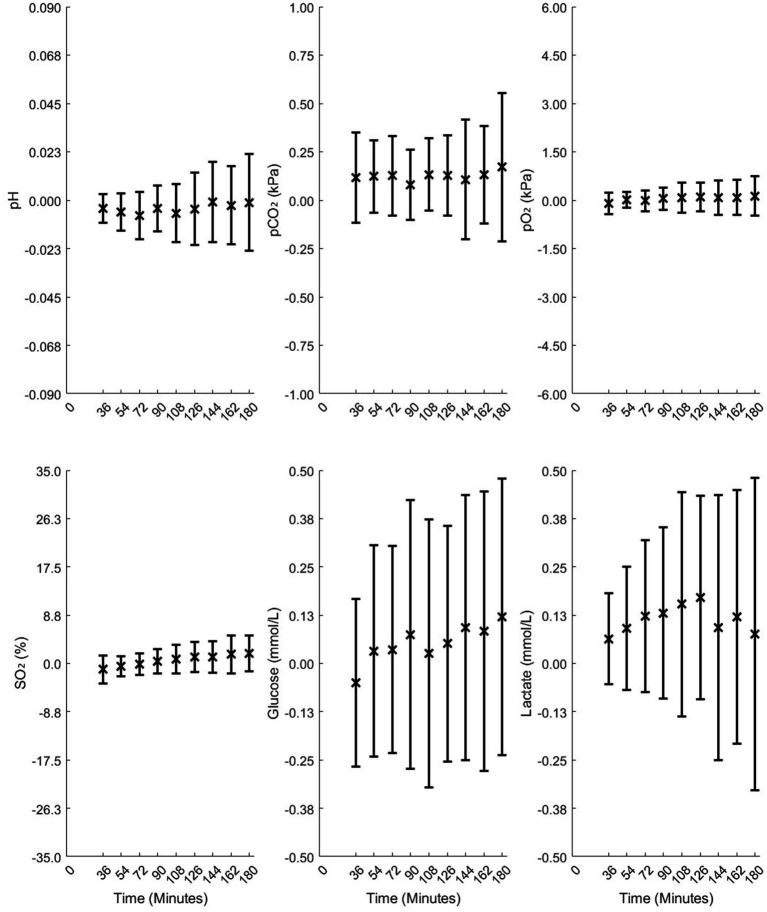
Mean differences and standard deviations (SD) of the differences between the measured and model-calculated values at sample times in standard blood gas syringes, calculated from time points of 36, 54, 72, 90, 108, 126, 144, 162, and 180 min in Study 1.

**Figure 4:**
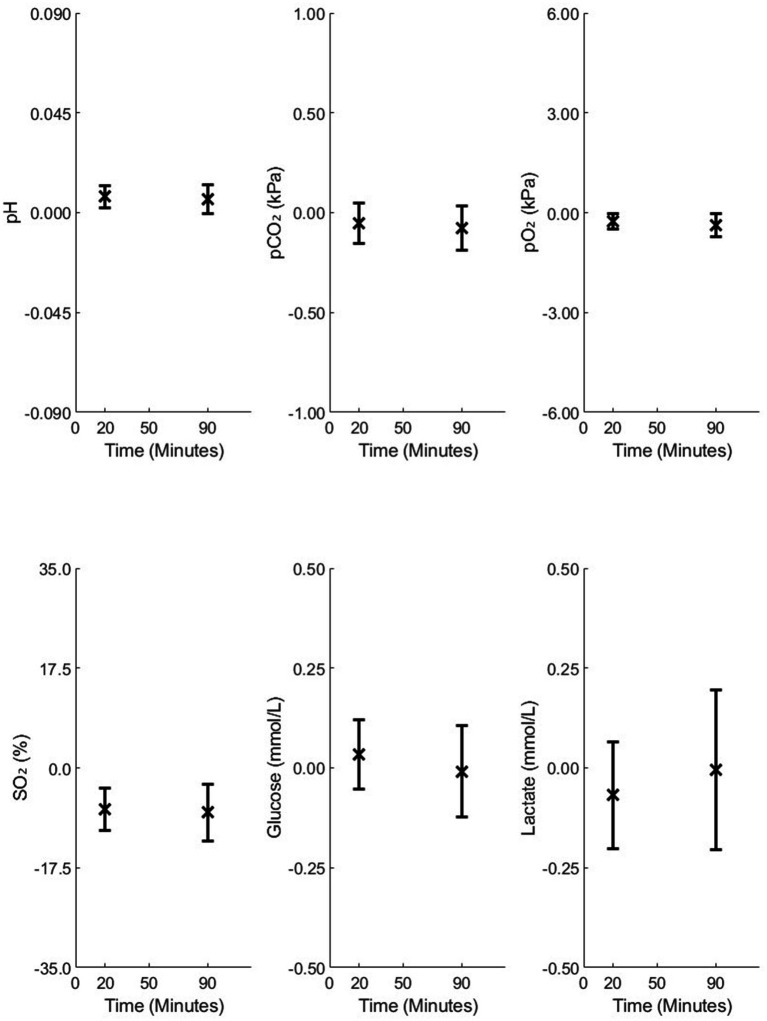
Mean differences and standard deviations (SD) of the differences between the measured and model-calculated values at the sample time, calculated from analysis time points of 20 and 90 min in 4-mL vacuum tubes in Study 2.

**Figure 5:**
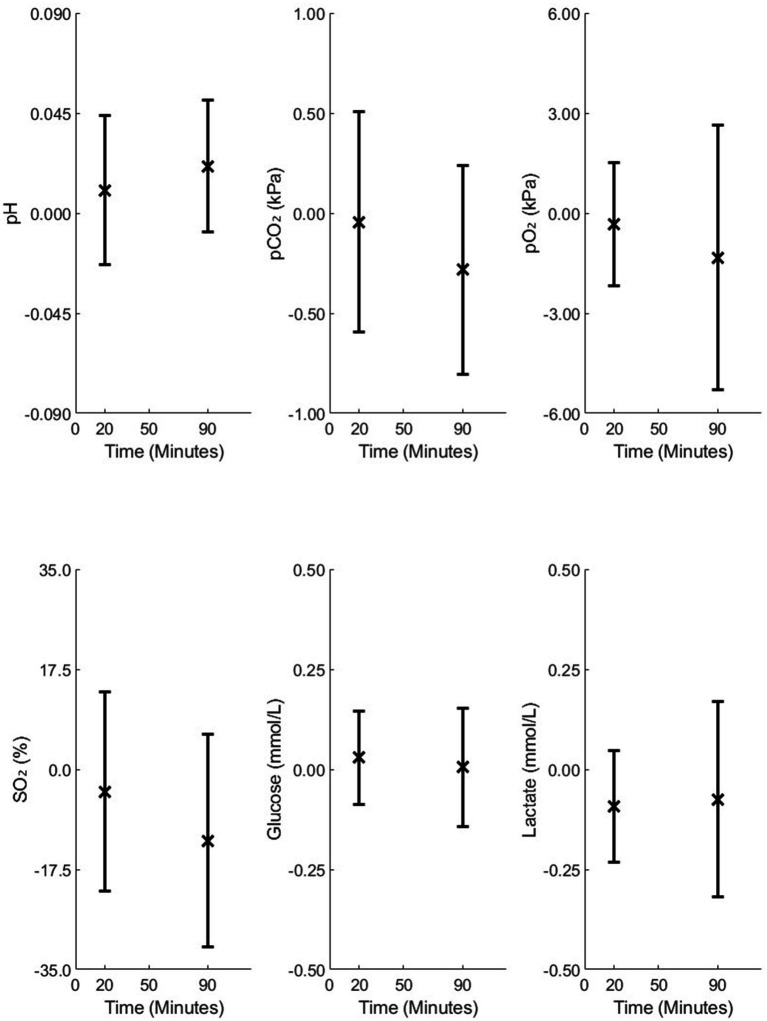
Mean differences and standard deviations (SD) of the differences between the measured and model-calculated values at the sample time, calculated from analysis time points of 20 and 90 min in 2-mL vacuum tubes in Study 2.

Figures 3–5 illustrate the mean differences (accuracy) and SD of the differences (precision) obtained by comparing measured and calculated values at the sample time from different analysis time points in Studies 1 and 2. Common to these figures are measurements taken 90 min after sampling, a duration that may be important in relation to blood sample transport to the laboratory. The mean and SD values at 90 min for Study 1 are as follows: pH = −0.004 ± 0.011, pCO₂ = 0.08 ± 0.18 kPa, pO₂ = 0.05 ± 0.34 kPa, SO₂ = 0.39 ± 2.21%, Glu = 0.07 ± 0.35 mmol/L, and Lac = 0.13 ± 0.22 mmol/L. For Study 2, the results at 90 min for 4-mL vacuum tubes are as follows: pH = 0.006 ± 0.007, pCO₂ = −0.07 ± 0.11 kPa, pO₂ = −0.37 ± 0.34 kPa, SO₂ = −7.79 ± 4.95%, Glu = 0.01 ± 0.11 mmol/L, and Lac = −0.00 ± 0.20 mmol/L. For 2-mL vacuum tubes, the results are as follows: pH = 0.021 ± 0.030, pCO₂ = −0.28 ± 0.52 kPa, pO₂ = −1.33 ± 3.96 kPa, SO₂ = −12.64 ± 18.27%, Glu = 0.00 ± 0.14 mmol/L, and Lac = −0.07 ± 0.24 mmol/L.

As illustrated in Figure 3, accuracy changed only slightly over 180 min for all variables except SO₂, while precision remained relatively stable up to 90 min. For Figures 4, 5, accuracy and precision changed only slightly between separate analyses at 20 and 90 min.

Comparison of standard blood gas syringes (Figure 3) and 4-mL vacuum tubes (Figure 4) with 2-mL vacuum tubes (Figure 5) shows that 2-mL tubes exhibit higher mean differences (lower accuracy) and higher SDs (lower precision) in all variables at both 20 and 90 min compared to the other two groups. The reason for this difference is addressed in the “Discussion” section.

## Discussion

In this study, we used a mathematical model to simulate acid-base, oxygenation, and metabolism in venous blood samples at the sample time, using measured values of blood samples analyzed at different times of analysis.

The results in standard blood gas syringes and 4-mL vacuum tubes illustrate that, for delays in the analysis up to 90 min, values describing acid-base, oxygenation, and metabolism at sample time can be accurately and precisely calculated. The differences between model-calculated and measured values of pH and pCO₂ at 90 min remain within the range previously reported for repeatability, where consecutive blood gas measurements from the same patient showed standard deviations of 0.01 for pH, 0.16 kPa for pCO₂, 0.15 kPa for pO₂, and 1.49% for SO₂ (15). Although the SD of differences between model-calculated and measured pO₂ values in standard blood gas syringes and 4-mL vacuum tubes was higher than Mallat’s SD (15), this is likely due to oxygen degradation occurring during storage. Our model substantially corrects the bias and variability introduced by storage, bringing the values closer to the true values measured at the sample time, as shown in Supplementary Figures 7, 10. However, the results from 4-mL vacuum tubes in Study 2 are better than those from standard blood gas syringes in Study 1, which can be attributed to greater Hb variability observed in Study 1. Thus, the residual variability reflects physiological and technical limits rather than model inadequacy, supporting the model’s validity for calculating pO₂ in stored samples from both standard blood gas syringes and 4-mL vacuum tubes. For vacuum tubes, pO₂ could be calculated accurately and precisely. However, because the pO₂ values were low (2–6 kPa), even small calibration errors in the oxygen dissociation curve led to large errors in the calculated SO₂, resulting in relatively poor accuracy for SO₂. This would not be expected at higher oxygenation levels. For SO₂ in Study 1, the bias did not decrease substantially, which could be attributed to variability in Hb present in this study. To accurately model the oxygen saturation curve, the model assumes stable Hb levels. Otherwise, shifts in the oxygen dissociation curve can make SO₂ estimation unreliable—even if pO₂ was well-modeled.

In addition, errors in pH and pCO_2_ at 90 min for standard blood gas syringes and 4-mL vacuum tubes were within those considered useful for clinical interpretation in clinical emergencies, i.e., 0.05 (95% CI 0.04–0.06) for pH and 0.88 (95% CI: 0.75, 1.01 kPa) for pCO_2_ reported by Rang et al. (16). Errors in calculated glucose and lactate were always within 0.5 mmol/L, even in the 2-mL vacuum tube data. These values were adequate to identify glucose abnormalities or hyperlactatemia.

The method presented in this study may therefore be useful in clinical practice and change current opinion as to the use of blood with analysis delay for acid-base status and oxygenation (1–9). Using blood following a delay of 90 min might improve the blood sampling logistics in both out-clinic and in-hospital situations. Samples could be taken at the start of an ambulance transport prior to the start of therapy, analyzed in the hospital, and compared to a newly drawn blood sample in order to evaluate prehospital treatment. Similarly, samples could be taken in the ward and analyzed centrally, reducing the need for decentralized point-of-care technology. Such an approach might be integrated into the clinical workflow, with the only additional data required being the delay duration and the type of tube used. In addition, the use of vacuum tubes may reduce the number of venous punctures, as samples could be taken together with those tubes for other purposes.

The same mathematical model has been applied here for the analysis of standard blood gas syringes, vacuum tubes, different delays, and both healthy subjects and critically ill patients. The model seems to be robust over a wide range of applications and patients. This, however, was not the case for calculations made in vacuum tubes with only 2 mL of blood. For vacuum tubes with a small blood volume—that is, a large gas volume—errors in calculating pO₂ and pCO₂ in blood during cooling result in large errors in the calculation of the mass of O₂ and CO₂ in the gas phase. This finding indicates that the method presented here is sensitive to vacuum tubes with large gas volumes. However, as shown in Supplementary Figure 9, these errors appear only in a small subset of subjects and are associated with poor Bland–Altman agreement, largely due to a few outliers. This reflects a numerical limitation of the current modeling approach, rather than a physiological inconsistency, and occurs only when corrected pO₂ values exceed ambient air levels. Further studies are required to understand the conditions resulting in these outliers, the identification of which may allow the use of the method in vacuum tubes with large gas volumes. However, it is encouraging that 4-mL vacuum tubes are the most widely used in clinical practice. In addition, the study is limited to only normal subjects in vacuum tubes and severely ill patients in standard blood gas syringes and limited to specific brands of syringes and vacuum tubes. Further analysis will be needed to assess the generalizability and limitations of the method.

The estimation of acid production (0.008 mmol/L/min) was derived from the dataset in Study 1, as published previously (11), and verified in the prior publication of Study 2 (12). However, this value can change under varying physiological conditions, including different hematocrit, glucose levels, oxygenation, temperature, and other patient-specific factors. While the fixed rate applied here provided good agreement across both critically ill patients in Study 1 and healthy subjects in Study 2, further research is required to assess model outcomes under broader physiological conditions.

We acknowledge that the cited values from Mallat and Rang (15, 16) reflect practical clinical thresholds for interpretation rather than statistical measures of variability, such as the smallest detectable difference (SDD). While these values provide useful context for clinical relevance, statistical benchmarks such as the SDD may offer additional value in future methodological assessments focused on test–retest reliability.

The applied mathematical model uses the exact time of sample analysis relative to collection. However, in routine clinical practice, sampling times were not always recorded with minute-level precision and may be approximate. Further studies are required to assess how this uncertainty could influence model outcomes.

## Conclusion

This study evaluated a method for calculating values at the sample time following delayed analysis in standard blood gas syringes and vacuum tubes. The results demonstrate that accurate and precise values can be obtained for syringes and certain vacuum tubes. This method may have clinical applications in improving the logistics of blood sampling and analysis and increasing the usability of blood samples acquired in out-of-hospital or prehospital settings, where analysis of drawn blood may take several hours.

## Data Availability

The data analyzed in this study is subject to the following licenses/restrictions: the dataset consists of anonymized patient data and is not publicly available due to ethical and privacy restrictions. Access to the data may be granted upon reasonable request and with appropriate institutional and ethical approvals. Requests to access these datasets should be directed to BN email: baharehn@hst.aau.dk.
