# Supplementary material for: Mathematical correction of the effects of storage time and gas contamination on blood sample measurements
Source: Front Med (Lausanne). 2025 Sep 25;12:1630871. doi: 10.3389/fmed.2025.1630871 (PMC12507878; doi:10.3389/fmed.2025.1630871)
Supplement: Supplementary file 1 [file Data_Sheet_1.PDF]

Mathematical Correction of the Effects of Storage Time and Gas Contamination on Blood Sample Measurements

Bahareh Nevirian[1]*, Lars Pilegaard Thomsen [1], Steen Kåre Fagerberg[3,4], Jette Nybo[2,3], Mette Krogh Pedersen[4], Kjeld Asbjørn Jensen Damgaard[4],Lisha Shastri[1], Søren Risom Kristensen[2,3], Stephen Edward Rees[1,*]

1. Respiratory and Critical Care (Rcare) Group, Aalborg University, Aalborg, Denmark
2. Department of Clinical Biochemistry, Aalborg University Hospital, Aalborg, Denmark
3. Department of Clinical Medicine, Aalborg University, Aalborg, Denmark
4. Anesthesia and Intensive Care Unit, Aalborg University Hospital, Aalborg, North Denmark Region, Denmark

*Corresponding author(s): e-mail: baharehn@hst.aau.dk, sr@hst.aau.dk

## Electronic supplementary material

This electronic supplementary material includes detailed explanations of the equations and a corresponding table of abbreviations, as well as Bland–Altman plots across all patients (Study 1) comparing measured and calculated values at the sample time, calculated from analysis times of 36, 54, 72, 90, 108, 126, 144, 162, and 180 minutes for pH, $pCO_2$, $pO_2$, $SO_2$, lactate, and glucose. In addition, it includes plots showing pre- and post-correction comparisons. For Study 2, the supplementary material includes Bland–Altman plots across all subjects for 4 mL and 2 mL vacuum tubes, with separate plots provided to show pre- and post-correction comparisons.

**<u>Detailed Explanation of the Equations</u>**

The model used in this and the previous studies (11,12) incorporates the mathematical model of the acid-base chemistry of the blood of Rees et al. (13,14) with some additions, as follows.

The mass balance equations, from 1 to 18 (Figure 1), address each component in the system, including $CO_2$, $O_2$, buffer base (BB), hemoglobin (Hb), glucose (Glu), and lactate (Lac). Equations 1 to 5 describe $CO_2$. Equation 1 describes the total concentration of $CO_2$ in the system ($\mathrm{tCO}_{2s}$) by considering concentrations in the blood ($\mathrm{tCO}_{2b}$) and gas compartments ($\mathrm{tCO}_{2g}$), weighted with their respective fractions in the blood ($f_b$) and gas phase ($f_g$). Equation 2 describes the concentration $CO_2$ in blood ($\mathrm{tCO}_{2b}$) by taking into account that in plasma ($\mathrm{tCO}_{2p}$) and in erythrocyte, ($\mathrm{tCO}_{2e}$), weighted by their fractions in plasma ($f_p$) and erythrocyte ($f_e$). Equation 3 describes total $CO_2$ in plasma, which is the sum of $CO_2$ dissolved ($\mathrm{CO}_{2p}$) and plasma bicarbonate ($HCO_{3p}^-$), and equation 4 describes the total $CO_2$ in erythrocyte by considering $CO_2$ dissolved, erythrocyte bicarbonate ($HCO_{3e}^-$), and $CO_2$ bound to deoxygenated and oxygenated hemoglobin ($HbNHCOO^-, HbO_2NHCOO^-$). Equation 5 is the definition of $CO_2$ concentration in the gas compartment ($\mathrm{tCO}_{2g}$), as the division of the mass of $CO_2$ ($\mathrm{mCO}_{2g}$) by the volume of gas ($\mathrm{V}_g$).

Equations 6 to 10 describe $O_2$. Equation 6 describes $O_2$ concentration in the system ($\mathrm{tO}_{2s}$) by considering the concentration in blood ($\mathrm{tO}_{2b}$) and gas compartments ($\mathrm{tO}_{2g}$), weighted with their respective fractions in the blood and gas ($f_b$, $f_g$), respectively. Equation 7 describes $O_2$ concentration in blood ($\mathrm{tO}_{2b}$) considering concentrations in plasma ($\mathrm{tO}_{2p}$) and erythrocyte ($\mathrm{tO}_{2e}$), weighted by the fractions in plasma ($f_p$)and erythrocytes ($f_e$), respectively. Equations

8 and 9 describe the total $O_2$ concentration in plasma ($tO_{2p}$) and erythrocytes ($tO_{2e}$). In plasma, $tO_2$ refers to the amount of physically dissolved $O_2$ (Equation 8), whereas in erythrocytes, in addition to the physically dissolved $O_2$, it is also bound to various forms of hemoglobin (Equation 9). Equation 10 is the definition of $O_2$ concentration in the gas compartment by dividing the mass ($mO_{2g}$) by the volume of gas ($V_g$).

Equation 11 describes the total concentration of buffer base in blood ($BB_b$) by accounting for that in plasma ($BB_p$) and erythrocytes ($BB_e$), weighted according to their respective fractions in plasma ($f_p$) and erythrocytes ($f_e$). Equation 12 describes base excess ($BE_b$) in blood, which is the difference between buffer base ($BB_b$) and normal buffer base ($nBB_b$) at $pCO_2$=5.3 kPa and pH=7.4. Equation 13 describes the concentration of BB in plasma ($BB_p$), as the sum of bicarbonate ($HCO_{3p}^-$) and non-bicarbonate buffer base ($A_p^-$). Equation 14 describes the concentration of BB in erythrocyte ($BB_e$) by considering bicarbonate ($HCO_{3e}^-$), side chain buffer sites on oxygenated and deoxygenated hemoglobin($Hb(R^-)_\beta$, $HbO_2(R^-)_\beta$) , the amino end of oxygenated and deoxygenated hemoglobin chains ($HbNH_2$, $HbO_2NH_2$) as well as those bound to $CO_2$ ($HbNHCOO^-$, $HbO_2NHCOO$).

Equations 15 and 16 describe the total hemoglobin in blood (Hb) by considering either (Equation 15) the different chemical forms of the amino end of the hemoglobin chains on oxygenated or deoxygenated hemoglobin($HbNH_3{}^+$, $HbNH_2$, $HbNHCOO^-$, $HbO_2NH_3{}^+$, $HbO_2NH_2$, $HbO_2NHCOO^-$), or (Equation 16) by accounting for the different chemical forms of the side chain buffers ($Hb(RH)_\beta$, $Hb(R^-)_\beta$, $HbO_2(RH)_\beta$, $HbO_2(R^-)_\beta$).

Equations 17 and 18 describe the total concentration of Glu and Lac, which is the sum of their concentrations in plasma ($Glu_p$ , $Lac_p$) and erythrocyte ($Glu_e$ , $Lac_e$), considering the respective fractions ($f_p, f_e$).

The model also includes mass action equations in blood to describe the buffering of hydrogen ions (Equations 19-27). These have all been described previously (14) and are briefly outlined here: the bicarbonate buffer system in plasma and erythrocyte (Equations 19,21), the non-bicarbonate buffer in plasma (Equation 20), the amino-end buffering on hemoglobin in de- oxygenated (Equation 22) and oxygenated forms (Equation 23), the binding of $CO_2$ to hemoglobin (Equations 24,25) and the buffering properties of amino acid-side chains (Equations 26,27).

Equation 28 is a representation of the oxygen dissociation curve with oxygen saturation calculated from partial pressure of oxygen ($pO_2$), pH, partial pressure of carbon dioxide ($pCO_2$), 2,3-Diphosphoglyceric acid (DPG), and temperature ($T_b$). The definition of saturation ($SO_{2b}$), considering concentrations of hemoglobin from either the amino ends or the side chains, are illustrated by Equations 29 and 30, respectively. No mass-action equations are present in the gas phase.

Equations are included to describe the gas phase, including the relationship between partial pressures ($\mathrm{pCO}_{2g}, \mathrm{pO}_{2g}$) and fractions ($\mathrm{FCO}_{2g}, \mathrm{FO}_{2g}$) of $CO_2$ and $O_2$ (Equations 31,32), the definition of volume ($\mathrm{VCO}_{2g}, \mathrm{VO}_{2g}$) (Equations 33,34) and mass of each gas ($\mathrm{mCO}_{2g}, \mathrm{mO}_{2g}$) (Equations 35,36), and the definition of gas density ($\varphi$) (Equation 37).

Physio-chemical properties include equations describing the solubility of $CO_2$ and $O_2$ in plasma ($\mathrm{O}_{2p}, \mathrm{CO}_{2p}$) (Equations 38,39 ) and in the erythrocyte ($\mathrm{O}_{2e}, \mathrm{CO}_{2e}$) (Equations 40,41), the definitions of the fractions of gas ($f_g$) and blood ($f_b$) (Equation 42), and plasma ($f_p$) and erythrocyte ($f_e$) (Equation 43), where erythrocyte fraction ($f_e$) is defined assuming a constant hemoglobin concentration in the erythrocytes of 21 mmol/l (Equation 44).

There are two interfaces in the model: the interface between blood and gas and the interface between plasma and erythrocyte. For the blood and gas interface, partial pressures of $CO_2$ and $O_2$ are related using two parameters, $f_{equib,CO_2}, f_{equib,O_2}$, which describe the fractional equilibration between blood and gas phase (Equations 45,46).

In the second interface, hydrogen ions and lactate in plasma and erythrocyte fractions are assumed to reach a Donnan equilibrium, the mathematical representation of which is based on the work of Funder and Wieth (15) (Equations 47,48). In addition, the concentration of glucose in plasma and erythrocyte are considered to be equivalent due to passive diffusion of glucose between these two compartments (16) (Equation 49).

## Acronyms and abbreviations

| Abbreviation | Full Term / Description |
|---|---|
| $CO_2$ | Carbon Dioxide |
| $O_2$ | Oxygen |
| $tCO_{2s}$ | Total $CO_2$ in the system |
| $tCO_{2b}$ | Total $CO_2$ in blood |
| $tCO_{2g}$ | Total $CO_2$ in gas phase |
| $tCO_{2p}$ | Total $CO_2$ in plasma |
| $tCO_{2e}$ | Total $CO_2$ in erythrocyte |
| $tO_{2s}$ | Total $O_2$ in the system |
| $tO_{2b}$ | Total $O_2$ in blood |
| $tO_{2p}$ | Total $O_2$ in plasma |
| $tO_{2e}$ | Total $O_2$ in erythrocyte |
| $f_b$ | Fraction in blood |
| $f_g$ | Fraction in gas phase |
| $f_p$ | Fraction in plasma |
| $f_e$ | Fraction in erythrocyte |
| $HCO_{3p}^-$ | Bicarbonate in plasma |
| $HCO_{3e}^-$ | Bicarbonate in erythrocyte |
| $H_b$ | Hemoglobin |
| $H_bNHCOO^-$ | $CO_2$ bound to deoxygenated hemoglobin (carbamate) |
| $H_bO_2NHCOO^-$ | $CO_2$ bound to oxygenated hemoglobin (carbamate) |
| $H_bNH_2$ | Amino-end of deoxygenated hemoglobin |
| $H_bO_2NH_2$ | Amino-end of oxygenated hemoglobin |
| $H_bNH_3^+$ | Protonated amino-end of deoxygenated hemoglobin |
| $H_bO_2NH_3^+$ | Protonated amino-end of oxygenated hemoglobin |
| $Hb(R^-)_\beta$ | Deoxygenated hemoglobin side chain buffer ($R^-$ form) |
| $HbO_2(R^-)_\beta$ | Oxygenated hemoglobin side chain buffer ($R^-$ form) |
| $Hb(RH)_\beta$ | Protonated deoxygenated hemoglobin side chain buffer |
| $HbO_2(RH)_\beta$ | Protonated oxygenated hemoglobin side chain buffer |
| $A_p^-$ | Non-bicarbonate buffer in plasma |
| BB | Buffer Base |
| $BB_b$ | Buffer Base in blood |
| $BB_p$ | Buffer Base in plasma |
| $BB_e$ | Buffer Base in erythrocyte |
| $BE_b$ | Base Excess in blood |
| $nBB_b$ | Normal Buffer Base in blood |
| $mCO_{2g}$ | Mass of $CO_2$ in gas phase |
| $mO_{2g}$ | Mass of $O_2$ in gas phase |
| $V_g$ | Volume of gas phase |
| $pCO_2$ | Partial Pressure of $CO_2$ |
| $pO_2$ | Partial Pressure of $O_2$ |
| $pCO_{2g}$ | Partial Pressure of $CO_2$ in gas phase |

| | |
|---|---|
| $pO_{2g}$ | Partial Pressure of $O_2$ in gas phase |
| $FCO_{2g}$ | Fraction of $CO_2$ in gas |
| $FO_{2g}$ | Fraction of $O_2$ in gas |
| $VCO_{2g}$ | Volume of $CO_2$ in gas |
| $VO_{2g}$ | Volume of $O_2$ in gas |
| $\varphi$ | Gas density |
| $O_{2p}$ | $O_2$ in plasma |
| $O_{2e}$ | $O_2$ in erythrocyte |
| $CO_{2p}$ | $CO_2$ in plasma |
| $CO_{2e}$ | $CO_2$ in erythrocyte |
| DPG | 2,3-Diphosphoglyceric acid |
| $T_b$ | Blood temperature |
| $SO_{2b}$ | Oxygen Saturation |
| $f_{equib,CO2}$ | Fractional equilibration of $CO_2$ between blood and gas |
| $f_{equib,O2}$ | Fractional equilibration of $O_2$ between blood and gas |
| $Glu_p$ | Glucose in plasma |
| $Glu_e$ | Glucose in erythrocyte |
| $Lac_p$ | Lactate in plasma |
| $Lac_e$ | Lactate in erythrocyte |

# Study 1

Bland-Altman plots of comparisons of calculations and measurements at sample time from all measurement times for all variables and all time points.

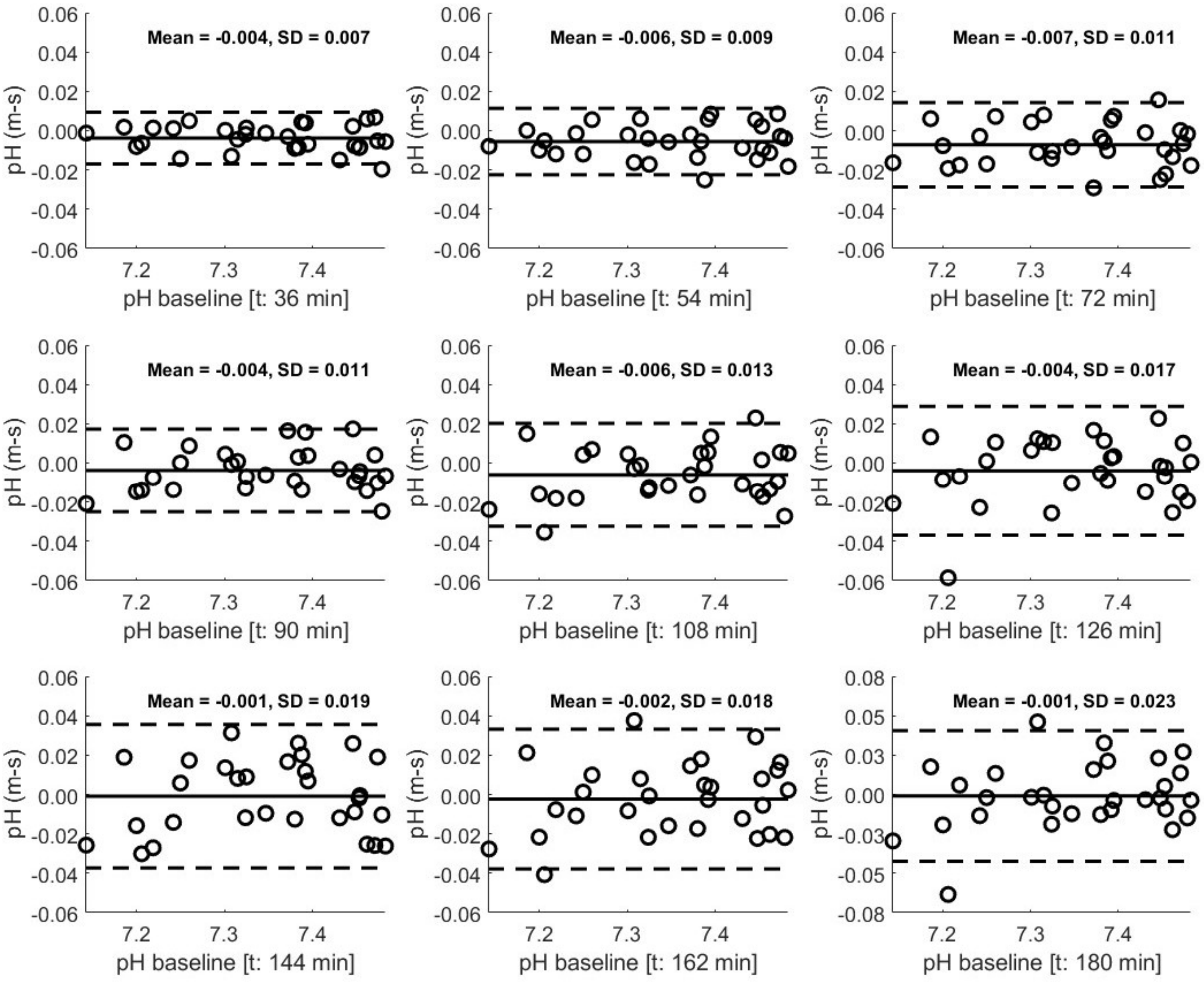

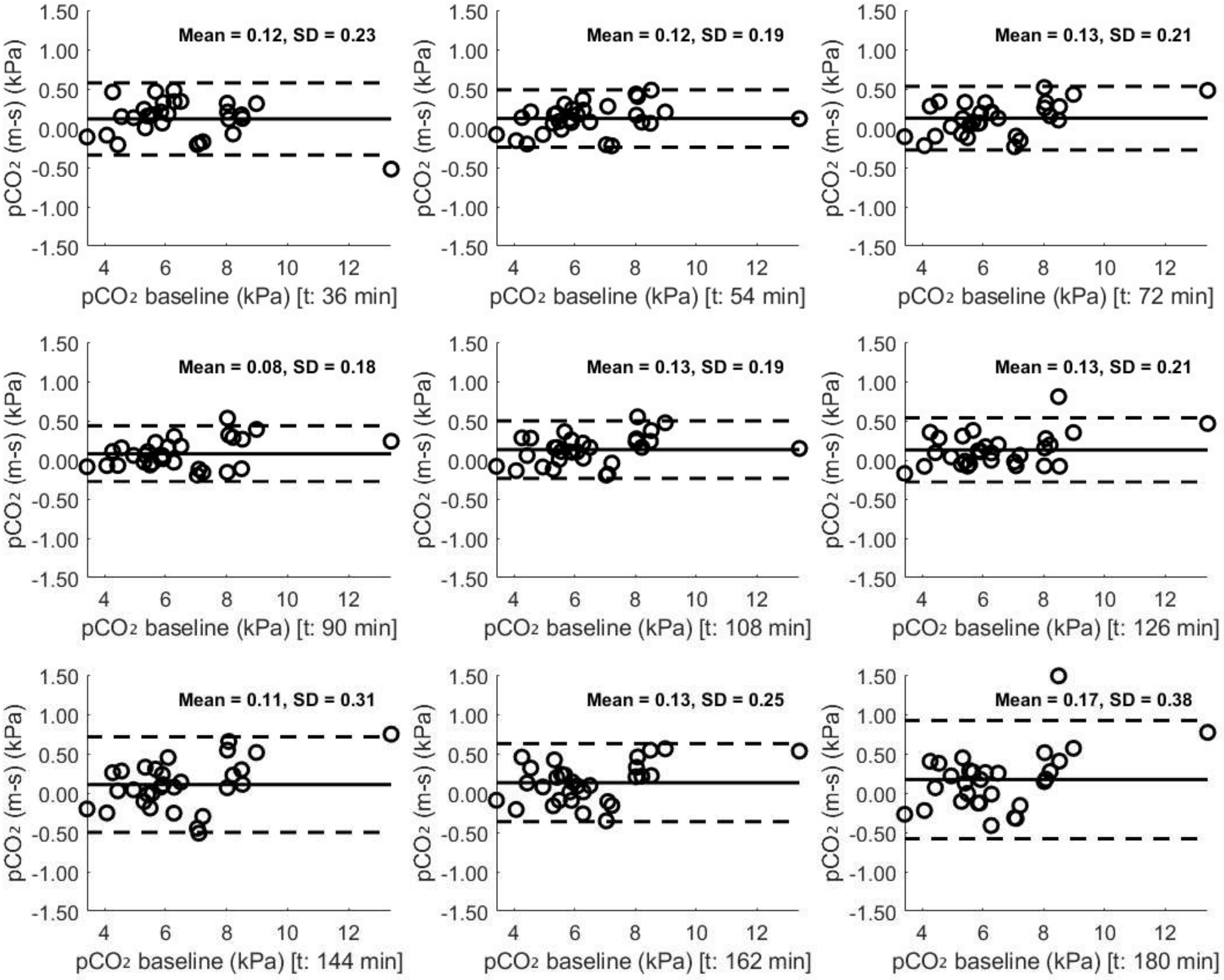
Mean = 0.12, SD = 0.23
pCO2 (m-s) (kPa)
pCO2 baseline (kPa) [t: 36 min]
Mean = 0.12, SD = 0.19
pCO2 (m-s) (kPa)
pCO2 baseline (kPa) [t: 54 min]
Mean = 0.13, SD = 0.21
pCO2 (m-s) (kPa)
pCO2 baseline (kPa) [t: 72 min]
Mean = 0.08, SD = 0.18
pCO2 (m-s) (kPa)
pCO2 baseline (kPa) [t: 90 min]
Mean = 0.13, SD = 0.19
pCO2 (m-s) (kPa)
pCO2 baseline (kPa) [t: 108 min]
Mean = 0.13, SD = 0.21
pCO2 (m-s) (kPa)
pCO2 baseline (kPa) [t: 126 min]
Mean = 0.11, SD = 0.31
pCO2 (m-s) (kPa)
pCO2 baseline (kPa) [t: 144 min]
Mean = 0.13, SD = 0.25
pCO2 (m-s) (kPa)
pCO2 baseline (kPa) [t: 162 min]
Mean = 0.17, SD = 0.38
pCO2 (m-s) (kPa)
pCO2 baseline (kPa) [t: 180 min]

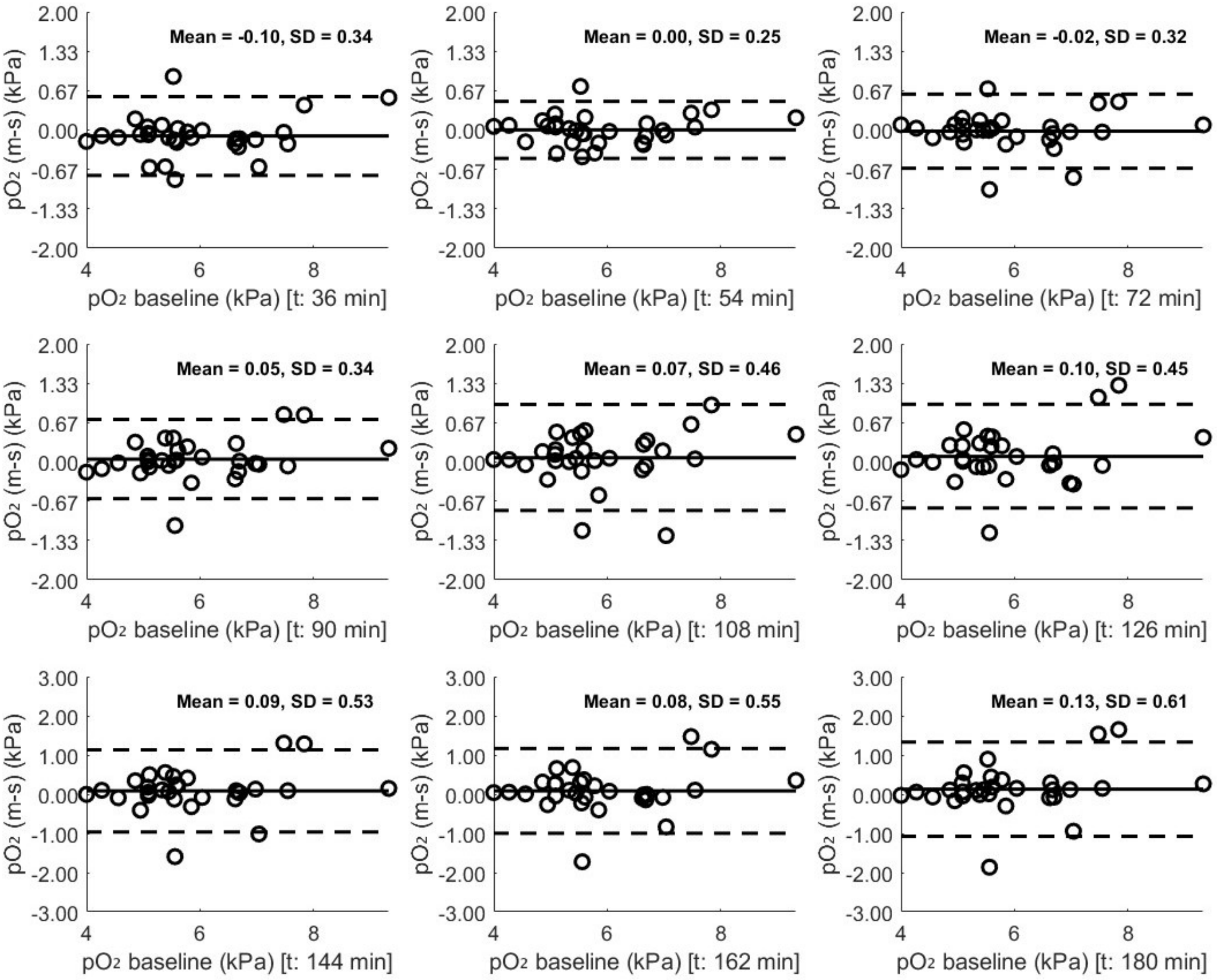
Mean = -0.10, SD = 0.34
Mean = 0.00, SD = 0.25
Mean = -0.02, SD = 0.32
Mean = 0.05, SD = 0.34
Mean = 0.07, SD = 0.46
Mean = 0.10, SD = 0.45
Mean = 0.09, SD = 0.53
Mean = 0.08, SD = 0.55
Mean = 0.13, SD = 0.61
pO2 (m-s) (kPa)
pO2 baseline (kPa) [t: 36 min]
pO2 baseline (kPa) [t: 54 min]
pO2 baseline (kPa) [t: 72 min]
pO2 baseline (kPa) [t: 90 min]
pO2 baseline (kPa) [t: 108 min]
pO2 baseline (kPa) [t: 126 min]
pO2 baseline (kPa) [t: 144 min]
pO2 baseline (kPa) [t: 162 min]
pO2 baseline (kPa) [t: 180 min]

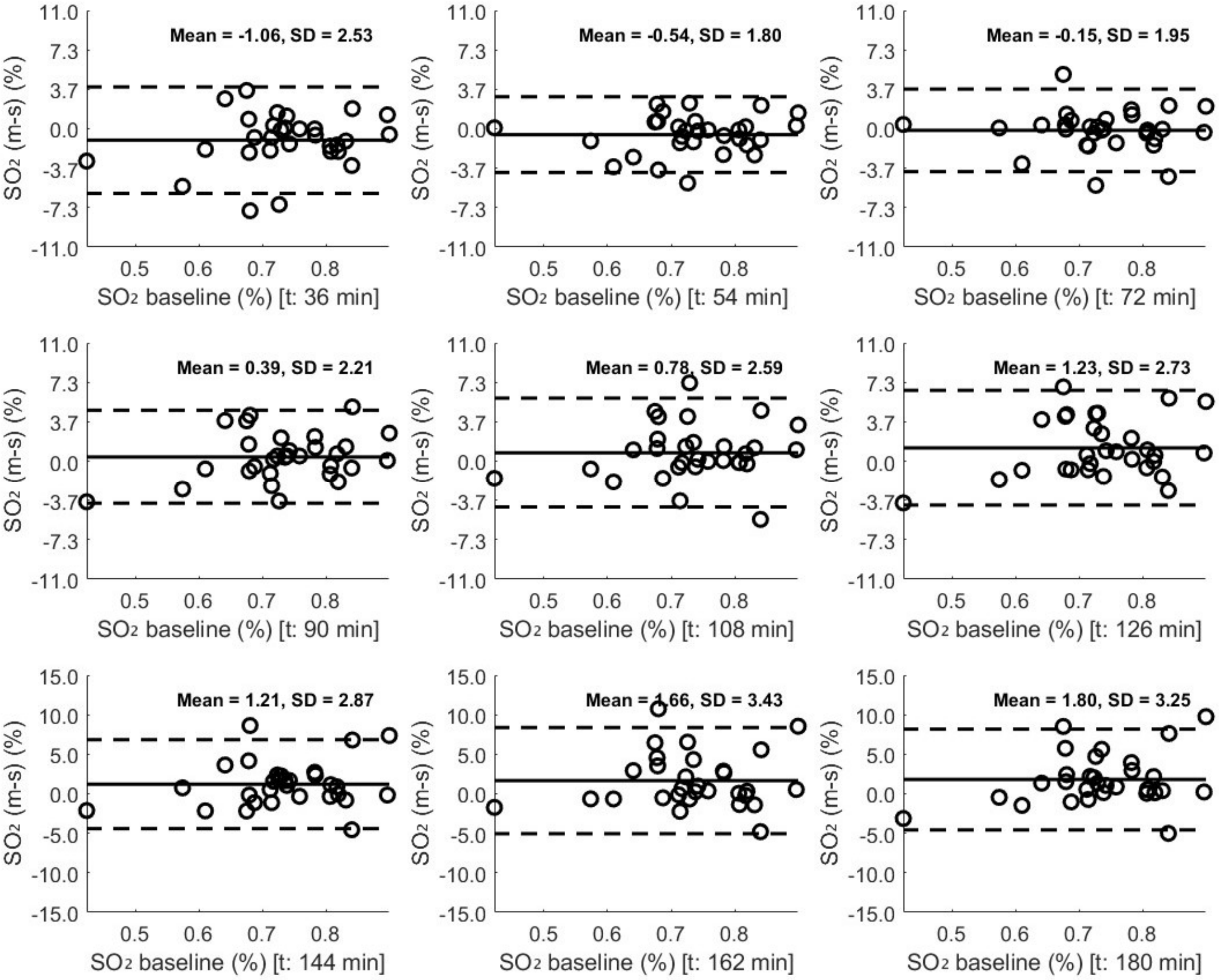

Mean = -1.06, SD = 2.53
Mean = -0.54, SD = 1.80
Mean = -0.15, SD = 1.95
Mean = 0.39, SD = 2.21
Mean = 0.78, SD = 2.59
Mean = 1.23, SD = 2.73
Mean = 1.21, SD = 2.87
Mean = 1.66, SD = 3.43
Mean = 1.80, SD = 3.25
SO2 (m-s) (%)
SO2 baseline (%) [t: 36 min]
SO2 baseline (%) [t: 54 min]
SO2 baseline (%) [t: 72 min]
SO2 baseline (%) [t: 90 min]
SO2 baseline (%) [t: 108 min]
SO2 baseline (%) [t: 126 min]
SO2 baseline (%) [t: 144 min]
SO2 baseline (%) [t: 162 min]
SO2 baseline (%) [t: 180 min]

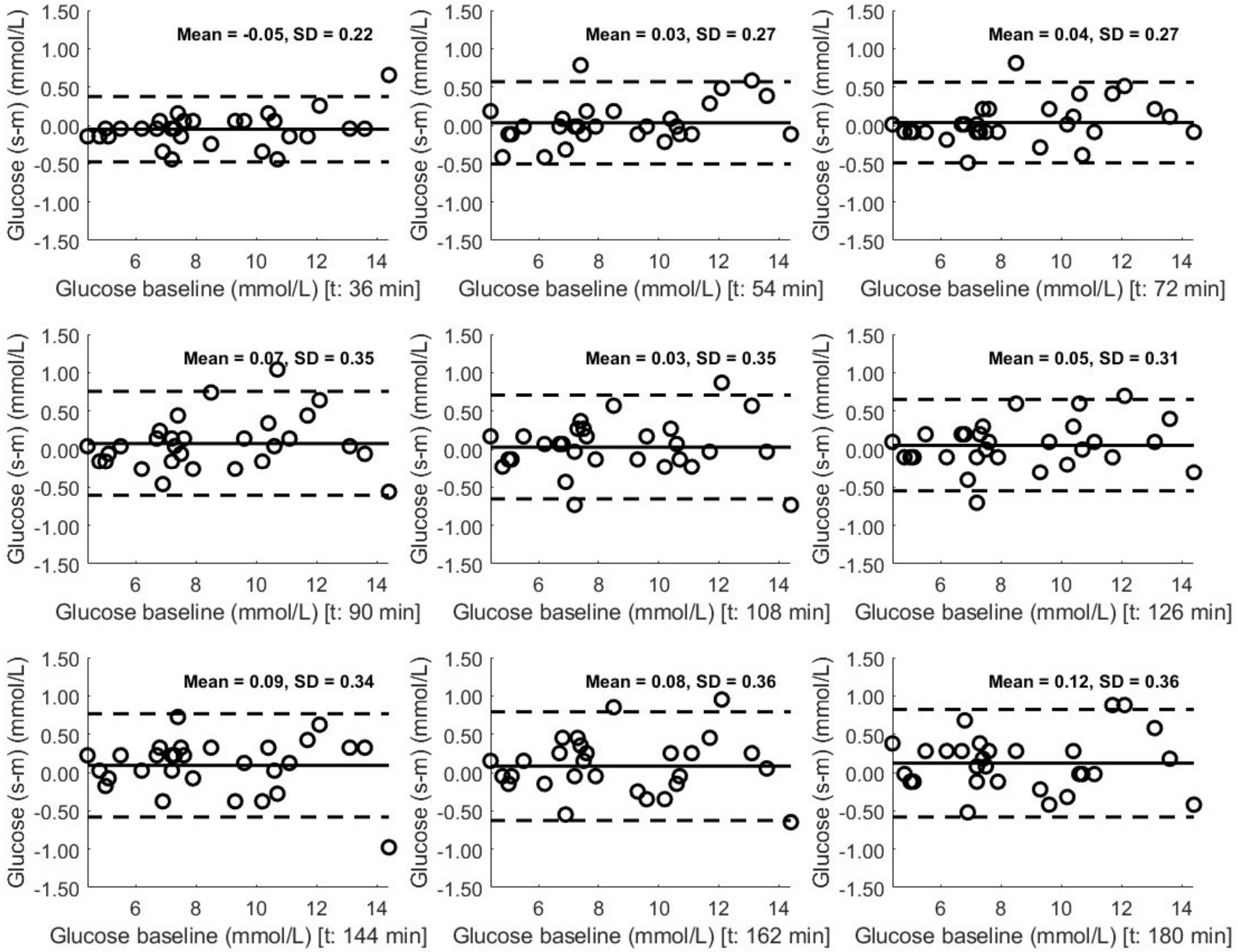
Mean = -0.05, SD = 0.22
Glucose baseline (mmol/L) [t: 36 min]
Mean = 0.03, SD = 0.27
Glucose baseline (mmol/L) [t: 54 min]
Mean = 0.04, SD = 0.27
Glucose baseline (mmol/L) [t: 72 min]
Mean = 0.07, SD = 0.35
Glucose baseline (mmol/L) [t: 90 min]
Mean = 0.03, SD = 0.35
Glucose baseline (mmol/L) [t: 108 min]
Mean = 0.05, SD = 0.31
Glucose baseline (mmol/L) [t: 126 min]
Mean = 0.09, SD = 0.34
Glucose baseline (mmol/L) [t: 144 min]
Mean = 0.08, SD = 0.36
Glucose baseline (mmol/L) [t: 162 min]
Mean = 0.12, SD = 0.36
Glucose baseline (mmol/L) [t: 180 min]
Glucose (s-m) (mmol/L)

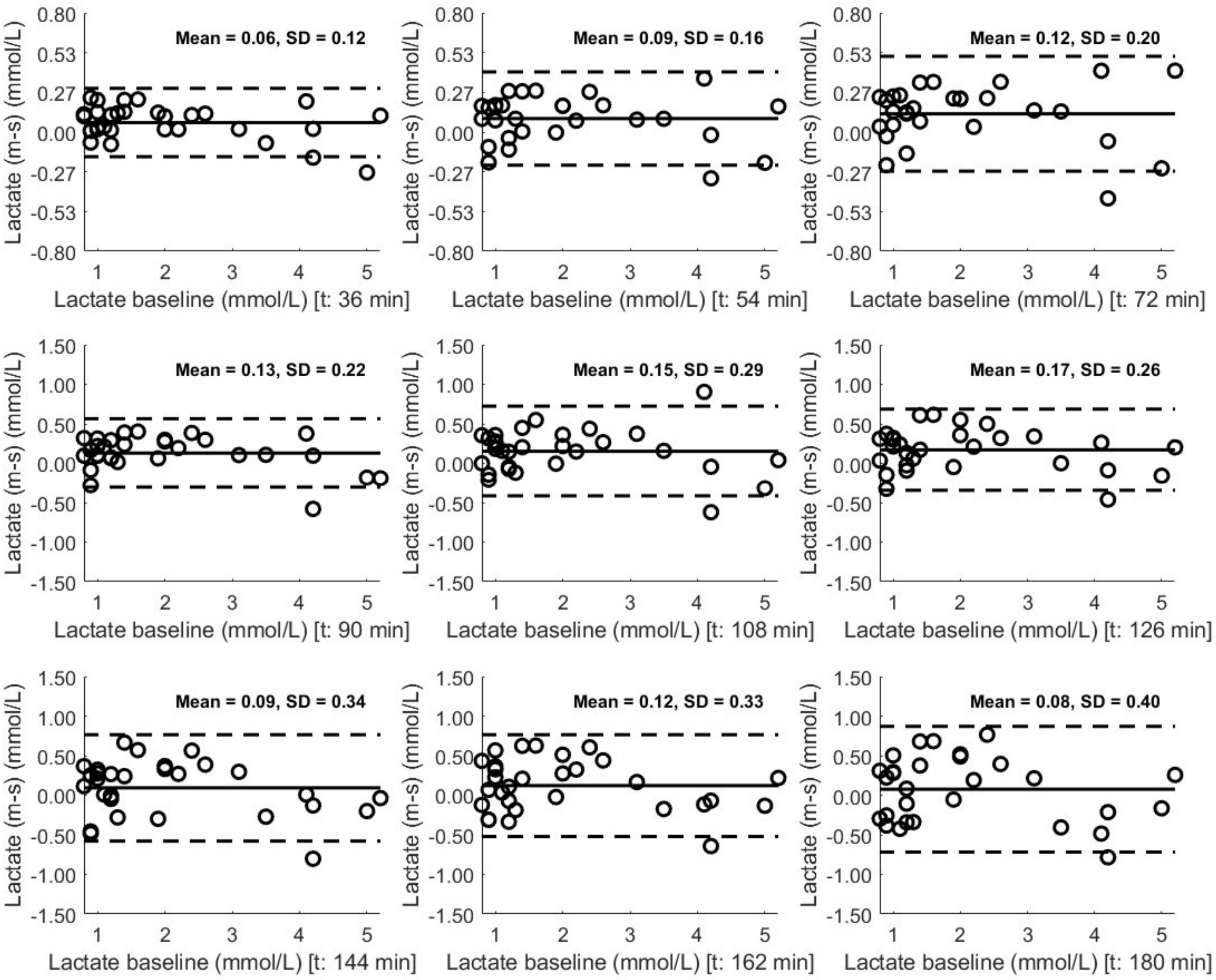


Figures 1 to 6 illustrate Bland–Altman plots comparing measured and model-simulated values at the sample time in standard blood gas syringes, calculated from analysis times of 36, 54, 72, 90, 108, 126, 144, 162, and 180 minutes across all patients in Study 1. m=measured; s=simulated

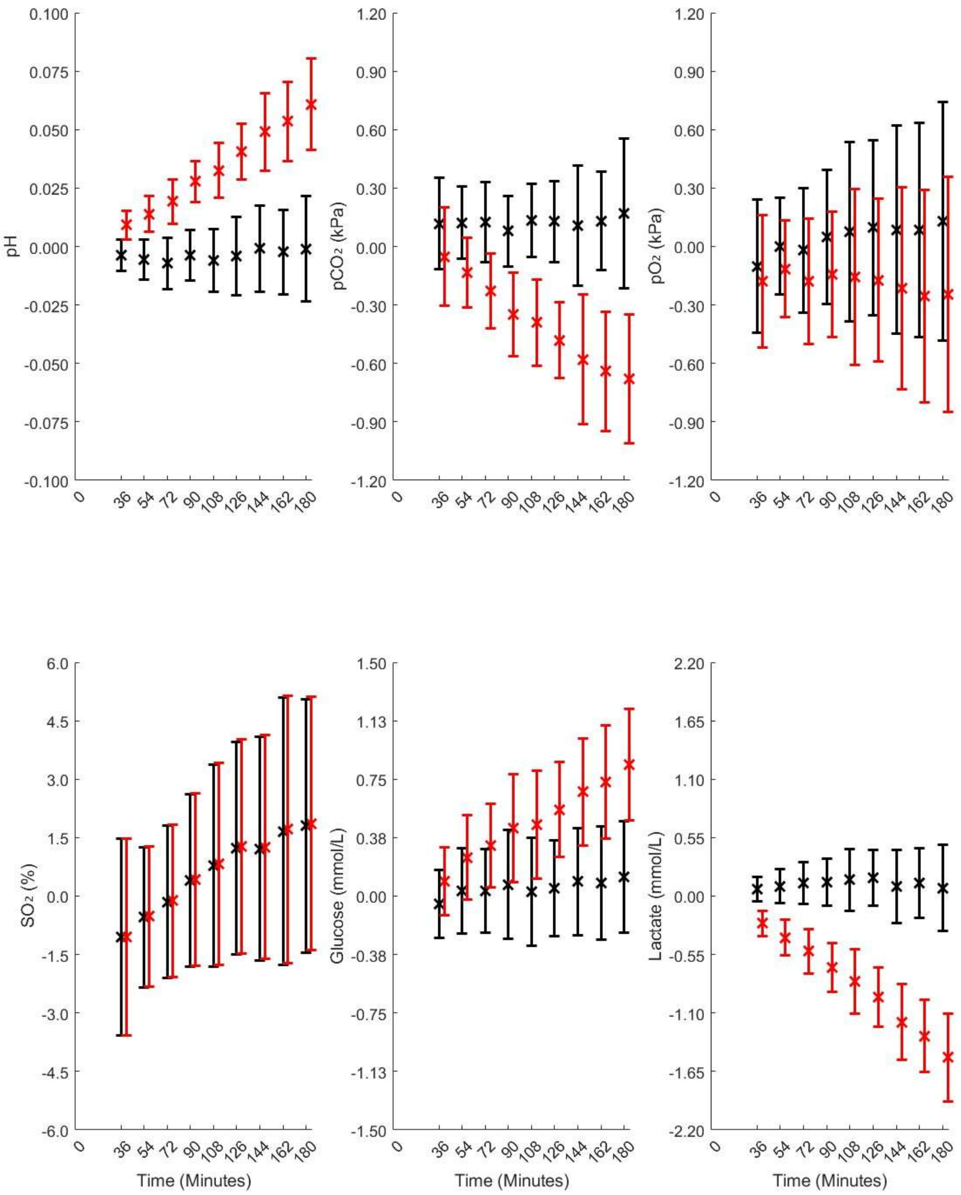


Figure 7 illustrates the comparison between pre-correction (measured vs. measured) and post-correction (measured vs. model-simulated), shown as mean ± standard deviation (SD) from samples collected in standard blood gas syringes in Study 1. Red lines represent pre-correction; black lines represent post-correction

## Study 2

In Study 2, a single mean difference and standard deviation (SD) of the differences between measured and model-simulated values at sample time is calculated using combined data from both the 20- and 90-minute time points, because the results at 20 minutes were similar to those at 90 minutes, showing only minimal differences.

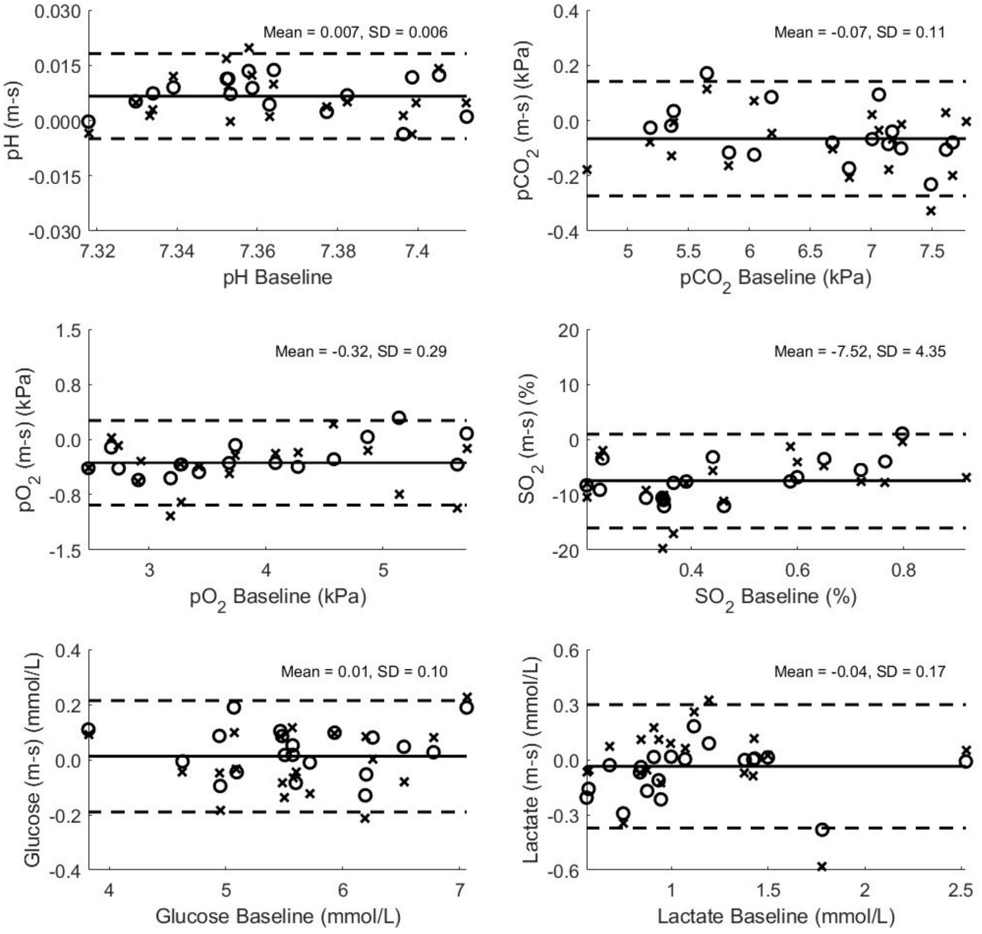


Figure 8: Bland-Altman plots comparing measured (m) and model simulated (s) values at sample time for all subjects for pH, $pCO_2$, $pO_2$, $SO_2$, lactate and glucose for 4 mL vacuum tubes. Circles (○) represent values simulated from measurements at 20 minutes, while crosses (×) represent values simulated from measurements at 90 minutes. The baseline is defined as the measured values of blood samples analyzed at sample time. Dashed lines represent the 95% limits of agreement (LoA).

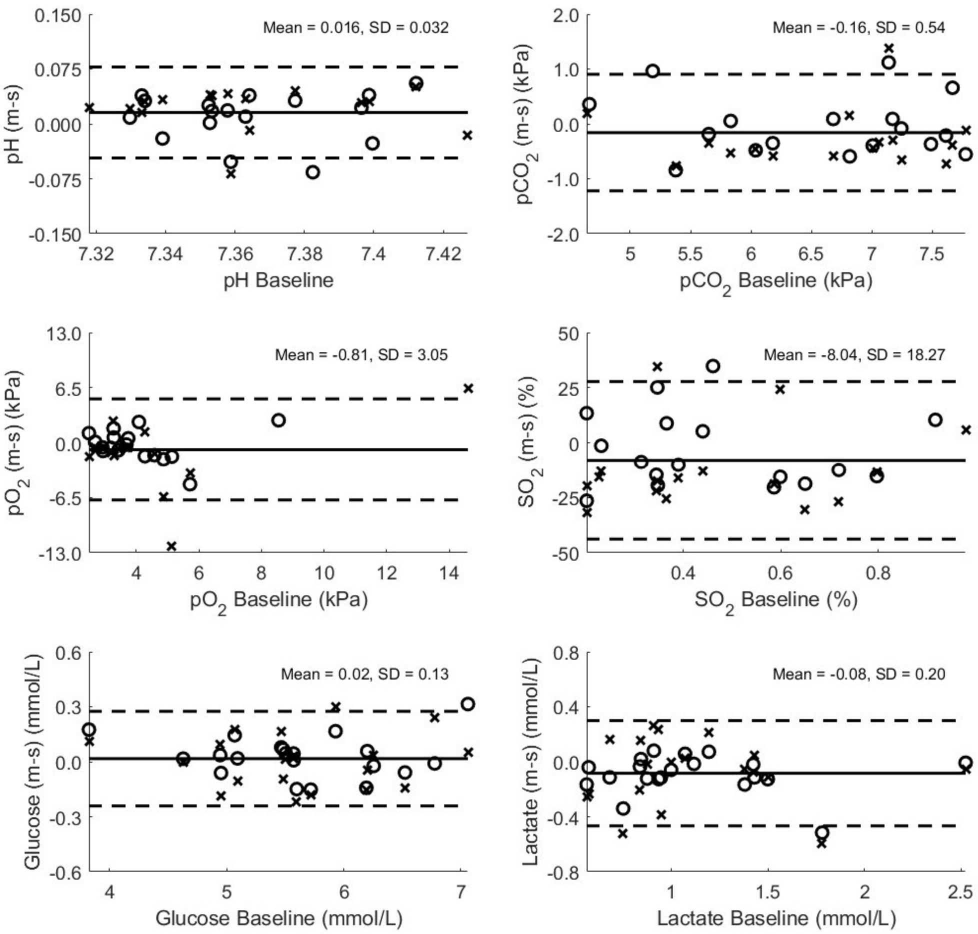


Figure 9: Bland-Altman plots comparing measured (m) and model simulated (s) values at sample time for all subjects for pH, $pCO_2$, $pO_2$, $SO_2$, lactate and glucose for 2 mL vacuum tubes. Circles (○) represent values simulated from measurements at 20 minutes, while crosses (×) represent values simulated from measurements at 90 minutes. The baseline is defined as the measured values of blood samples analyzed at sample time. Dashed lines represent the 95% limits of agreement (LoA).

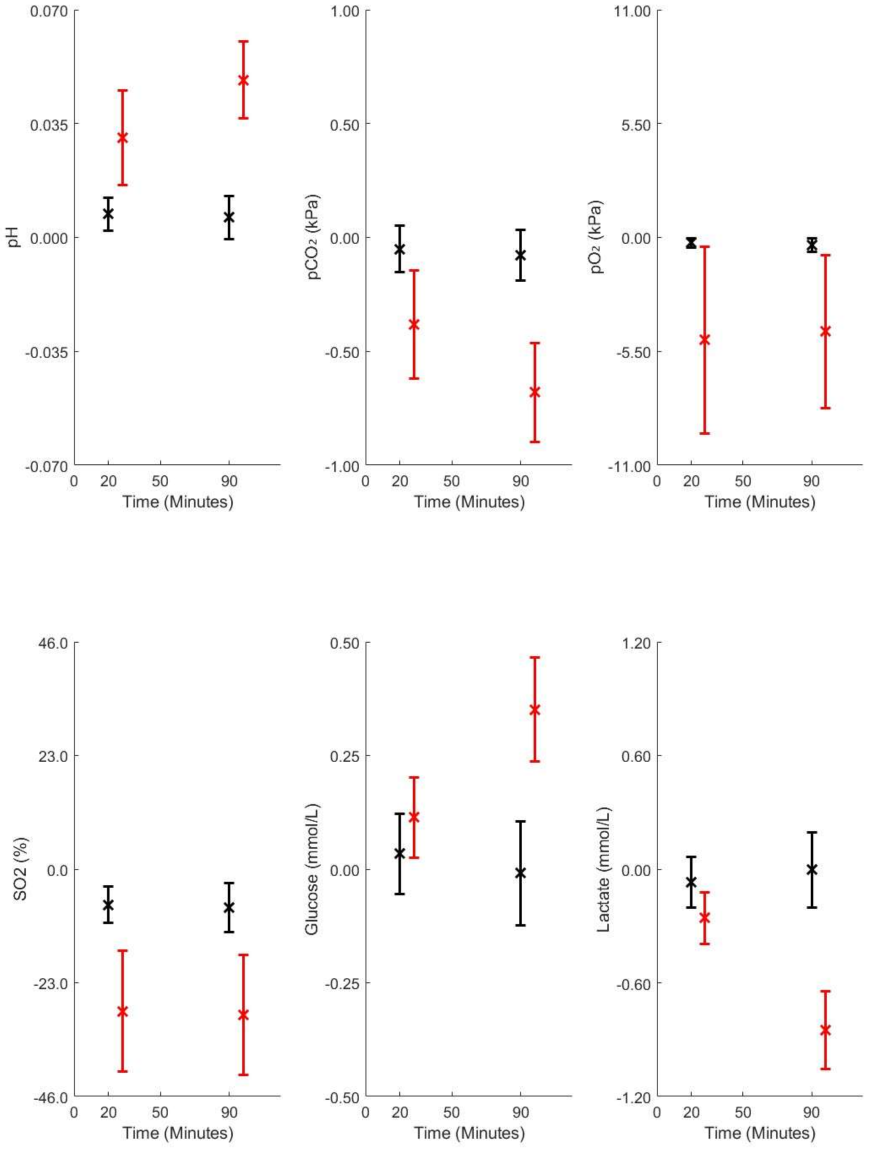


Figure 10 illustrates the comparison between pre-correction (measured vs. measured) and post-correction (measured vs. model-simulated), shown as mean ± standard deviation (SD) from samples collected in 4 mL vacuum tubes in Study 2. Red lines represent pre-correction; black lines represent post-correction

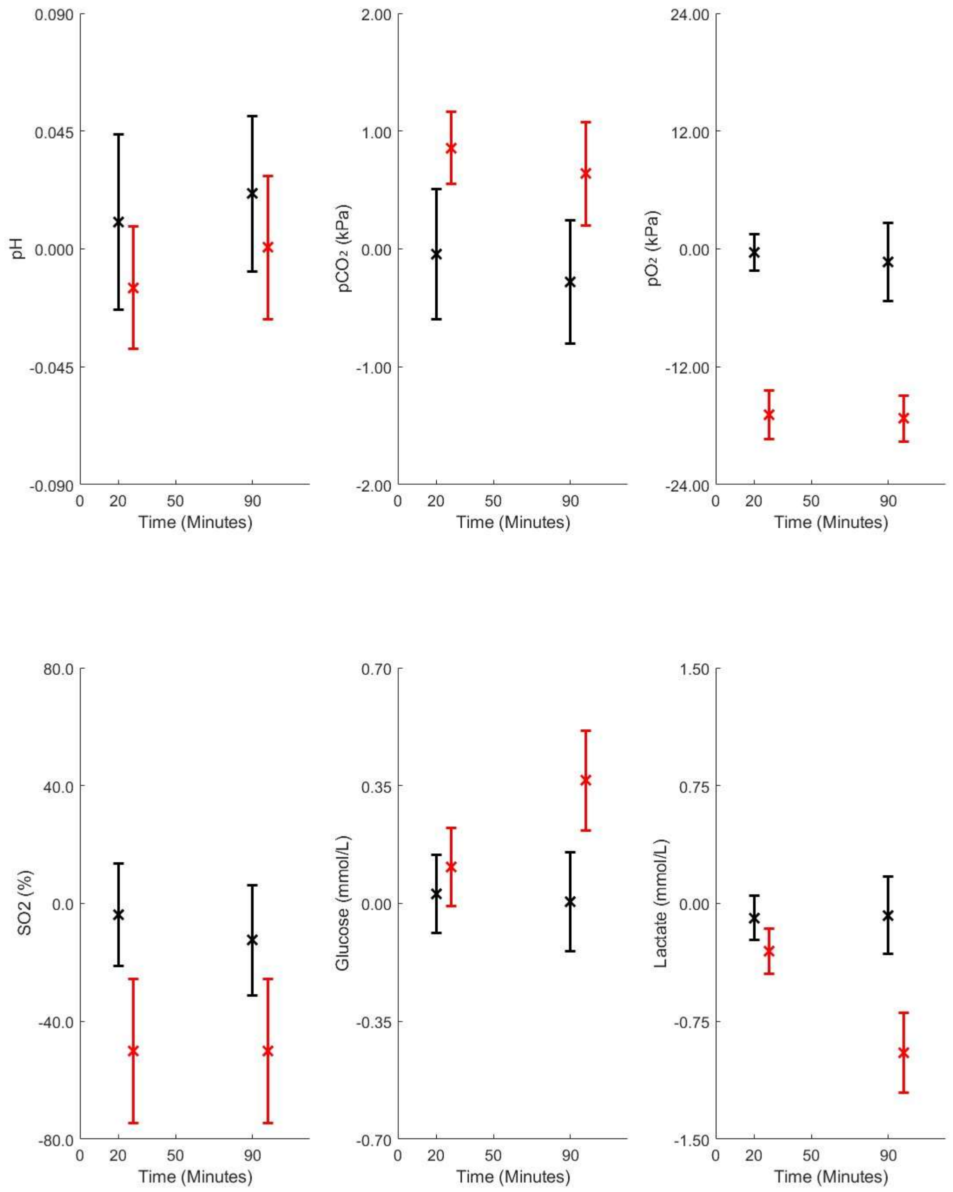


Figure 11 illustrates the comparison between pre-correction (measured vs. measured) and post-correction (measured vs. model-simulated), shown as mean ± standard deviation (SD) from samples collected in 2 mL vacuum tubes in Study 2. Red lines represent pre-correction; black lines represent post-correction
